# Liver Fibrosis Markers Represent Central Venous Pressure in Post-pubertal Patients With Congenital Heart Disease

**DOI:** 10.7759/cureus.39845

**Published:** 2023-06-01

**Authors:** Hideharu Oka, Kouichi Nakau, Yuki Shibagaki, Keita Ito, Yuki Sasaki, Rina Imanishi, Sorachi Shimada, Satoru Takahashi

**Affiliations:** 1 Pediatrics, Asahikawa Medical University, Asahikawa, JPN

**Keywords:** type iv collagen 7s, central venous pressure, liver fibrosis marker, hepatic congestion, congenital heart disease (chd)

## Abstract

Background

Central venous pressure (CVP) is one of the most important hemodynamic parameters in patients with congenital heart disease (CHD). In adults, it is well-known that liver fibrosis markers reflect CVP, but this is not well-understood in children. We investigated the liver fibrosis markers in pediatric CHD patients and their ability to predict CVP.

Methods

We studied 160 patients who underwent cardiac catheterization in our hospital between January 2017 and December 2020. The levels of the fibrotic markers, including type IV collagen 7s, procollagen type III peptide, and hyaluronic acid, were measured.

Results

Procollagen type III peptide was markedly elevated in infants younger than one year of age. From one to 15 years of age, it was slightly lower than in the infant group, with a peak at around 10 years of age. In the age group of 16 years and older, most of its values were generally high. Type IV collagen 7s and hyaluronic acid levels were high in infants, with no significant differences at later ages. Procollagen type III peptide and hyaluronic acid showed no significant correlation with CVP in any of the age groups, whereas type IV collagen 7s significantly correlated with CVP in the age group above one year old.

Conclusions

We found that elevated liver fibrosis markers, particularly type IV collagen 7s, correlated with central venous pressure in CHD patients older than one year. Measurement of liver fibrosis markers may allow the early detection of changes in CVP and liver function in patients with CHD.

## Introduction

In patients with congenital heart disease (CHD), it is important to know the hemodynamic status of the right ventricle, especially the central venous pressure (CVP), which is a good indicator of systemic venous congestion. When CVP increases, blood flow stasis occurs in various organs. The liver is particularly susceptible to congestion, in addition to decreased perfusion and hypoxia. In patients who have undergone the Fontan procedure, hepatomegaly and liver dysfunction occur in one-third to one-half of patients, and cirrhosis occurs in about 26% [1]. Patients with biventricular repair may also experience liver dysfunction if congestion is severe. Although magnetic resonance imaging and ultrasound with Doppler are useful to evaluate hepatic congestion [2], it is also possible to easily identify it by using liver fibrosis markers.

The use of liver fibrosis markers to estimate CVP has been mainly reported in adults, and there are few reports on their usefulness for this indication in children [3-5]. Direct measurement of CVP requires cardiac catheterization, which is an invasive procedure. Therefore, CVP estimation using liver fibrosis markers can be very useful in terms of medical costs and patient burden. However, it is known that liver fibrosis markers fluctuate greatly with age, even in healthy children, because of collagen production during growth [6-9]. In children with CHD-related hemodynamic abnormalities, such fluctuations may limit the usefulness of these markers in predicting CVP.

We investigated the liver fibrosis markers in pediatric patients with CHD as well as their ability to predict CVP.

## Materials and methods

**Figure 1:**
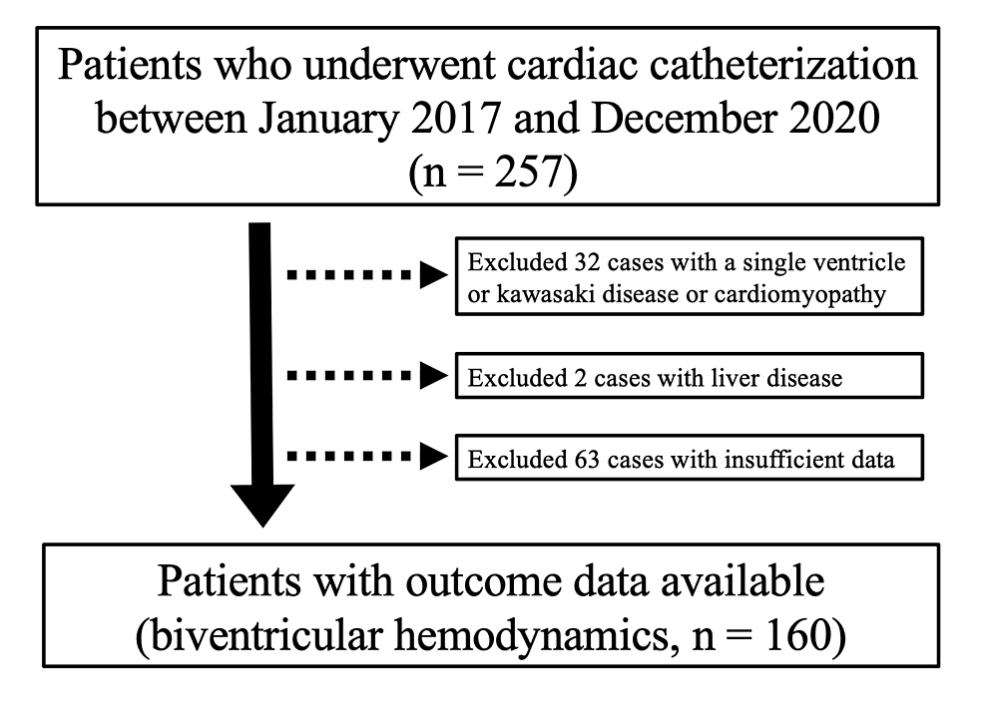
Patient flow

We conducted a retrospective study that included patients with CHD who underwent cardiac catheterization in Asahikawa Medical University Hospital, Hokkaido, Japan, between January 2017 and December 2020. A total of 257 patients were diagnosed with CHD. No cases of diabetes were found upon medical examination or review of laboratory results. We excluded 21 patients with a single ventricle to avoid the effects of hepatic congestion due to Fontan circulation, eight patients with Kawasaki disease, three patients with cardiomyopathy, two patients with liver diseases, such as biliary atresia or viral hepatitis, and 63 patients with insufficient data. We included 160 patients with biventricular hemodynamic data before and after surgery (Figure 1).

The breakdown of patients with heart disease was as follows: 31 patients had a ventricular septal defect, 25 patients had an atrial septal defect, 16 patients had tetralogy of Fallot, 15 patients had double outlet right ventricle, 10 patients had coarctation of the aorta, nine patients had aortic valve stenosis, nine patients had pulmonary atresia, eight patients had transposition of the great artery, eight patients had total anomalous pulmonary venous return (supracardiac type 3, cardiac type 2, infracardiac type 3), eight patients had pulmonary stenosis, seven patients had patent ductus arteriosus, five patients had an atrioventricular septal defect, two patients had Ebstein’s anomaly, two patients had coronary artery fistula, one patient had truncus arteriosus, one patient had interruption of the aortic arch, one patient had tricuspid stenosis, one patient had mitral valve prolapse, and one patient had mitral valve stenosis. We obtained written informed consent or assent from the patients or their parents. The patients underwent cardiac catheterization for hemodynamic parameter evaluation as part of routine follow-up or surgical planning. Cardiac catheterization was performed under the same anesthesia protocol for all patients, which included intravenous infusion of thiopental, midazolam, and dexmedetomidine. Ventricular volumes were calculated from biplane cine-angiocardiograms and expressed as a percentage of the normal values [10]. Right atrial pressure was used as a surrogate for CVP. Four groups were created by stratifying the CVP by 10 mmHg and the cardiac index (CI) by 3 L/min/m^2^. To evaluate the fibrotic status of the liver, the fibrotic markers procollagen type III peptide (PⅢP), type IV collagen 7s (4COL7s), and hyaluronic acid (HA) were measured by chemiluminescent enzyme immunoassay in addition to general biochemistry. Blood tests were performed within three days before cardiac catheterization. Normal values for liver fibrosis markers are known only in adults. Normal values are 3.6-9.5 ng/mL for PⅢP, <6 ng/mL for 4COL7s, and <50 ng/mL for HA.

All parameters are expressed as mean ± standard deviation values. The Shapiro-Wilk normality test was used to check the normal distribution of the data. Statistical differences were determined using the Pearson correlation and the Kruskal-Wallis test. Statistical significance was set at p <0.05. Statistical calculations were performed using the Statistical Package for the Social Sciences (version 24.0; IBM Corp., Armonk, NY). This study was conducted in compliance with the standards of the Declaration of Helsinki and the current ethical guidelines and was approved by our institutional ethics board (No. 19229).

## Results

 Table 1 shows the demographic characteristics and hemodynamic and laboratory data.

**Table 1:** Demographic characteristics and data from hemodynamic and laboratory tests Data are given as mean and standard deviation. CVP = central venous pressure; RVP = right ventricular pressure; LVP = left ventricular pressure; LVOTS = left ventricular outflow tract stenosis pressure gradient; %RVEDV = % of normal right ventricular end-diastolic volume; %LVEDV = % of normal left ventricular end-diastolic volume; Plt = platelet; T-bil = serum total bilirubin; AST = serum aspartate aminotransferase; ALT = serum alanine aminotransferase; γGTP = serum γ-glutamyl transpeptidase; PⅢP = procollagen type Ⅲ peptide; 4COL7s = type Ⅳ collagen 7s; HA = hyaluronic acid; ACEi = angiotensin-converting-enzyme inhibitor; ARB = angiotensin II receptor blocker

	< 1 years (n = 18)	1 - 9 years (n = 86)	10 - 15 years (n = 28)	≧ 16 years (n = 28)
CVP (mmHg)	4.2 ± 1.7	4.3 ± 1.7	5.6 ± 2.5	8.0 ± 3.8
RVP/LVP	0.75 ± 0.27	0.44 ± 0.26	0.39 ± 0.29	0.54 ± 0.78
LVOTS (mmHg)	6.0 ± 9.3	3.8 ± 8.1	9.4 ± 20.4	2.8 ± 7.0
%RVEDV	124.8 ± 22.7	125.9 ± 30.4	119.0 ± 27.7	102.3 ± 30.7
%LVEDV	129.6 ± 57.3	113.4 ± 24.2	109.9 ± 21.8	116.4 ± 25.4
Cardiac Index (L/min/m^2^)	4.6 ± 0.9	4.3 ± 1.1	3.8 ± 0.7	3.1 ± 0.8
Plt (×10^4^/μL)	43.0 ± 12.8	32.7 ± 9.8	22.9 ± 7.0	19.4 ± 6.4
T-bil (mg/dL)	0.4 ± 0.1	0.6 ± 0.2	0.9 ± 0.4	0.9 ± 0.4
AST (U/L)	46.4 ± 14.8	31.5 ± 7.5	24.1 ± 4.0	24.6 ± 9.8
ALT (U/L)	27.2 ± 17.1	16.2 ± 11.1	14.4 ± 5.2	21.0 ± 11.7
γGTP (U/L)	14.2 ± 4.2	12.9 ± 5.2	17.6 ± 6.3	32.8 ± 27.7
PIIIP (ng/mL)	112.0 ± 57.1	33.0 ± 16.0	36.5 ± 13.9	12.4 ± 4.8
4COL7s (ng/mL)	8.3 ± 1.4	5.2 ± 1.3	5.5 ± 1.0	5.8 ± 1.6
HA (ng/mL)	28.4 ± 8.8	16.3 ± 8.1	16.2 ± 9.1	21.8 ± 18.9
Medication: ACEi/ARB (number of patients)	2	2	4	4

There was no significant difference in CVP among the age group below 15 years, whereas it was significantly higher in the age group above 16 years than in the group below nine years (p < 0.001). The right ventricular pressure-left ventricular pressure ratio was significantly higher in the age group of one year than in the other groups (p < 0.01). The left ventricular outflow tract pressure gradient was not significantly different among the groups. With respect to ventricular volumes, the right ventricular volume was significantly lower in the age group above 16 years than in the group below nine years (p < 0.05). The left ventricular volume was not significantly different among the groups. CI was not significantly different among the age group below 15 years but was significantly lower in the age group above 16 years than in the other groups (p < 0.05). The general laboratory tests of liver biochemistry showed age-related variations, with very rare abnormalities, and no significant differences were observed above 10 years of age. The level of PⅢP was significantly higher in the age group under one year than in the other age groups (p <0.01), and there was no significant difference in the age group of 1-15 years. Both 4COL7s and HA levels were significantly higher in patients younger than one year of age (p <0.01), with no significant difference among the other age groups. There was no significant difference in the number of patients using angiotensin-converting-enzyme inhibitor/angiotensin II receptor blockers.

**Figure 2:**
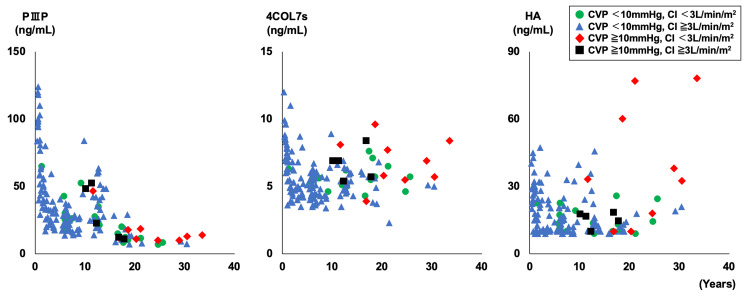
Liver fibrosis markers for each age Green circles are CVP less than 10 mmHg and CI less than 3 mL/min/m^2^, blue triangles are CVP less than 10 mmHg and CI more than 3 mL/min/m^2^, red diamonds are CVP more than 10 mmHg and CI less than 3 mL/min/m^2^, black squares are CVP more than 10 mmHg and CI more than 3 mL /min/m^2^. Three patients with abnormally high PIIIP (199, 208, 269ng/mL) were excluded from the graph. CVP: central venous pressure, CI: cardiac index, PIIIP: procollagen type III peptide, 4COL7s: type IV collagen 7s, HA: hyaluronic acid

Figure 2 shows the PⅢP, 4COL7s, and HA values for all age groups, classified by CVP and CI.

The PⅢP level was higher in the younger age group and markedly higher in the group comprising children below one year of age. From one to 15 years of age, PⅢP values were slightly lower than those at one year of age but were clearly higher than the normal values for adults, with a peak at around 10 years of age. In the age group of 16 years and older, there were several cases within the normal adult PⅢP range, but most cases were generally high. High CVP levels were more common in patients over 16 years of age. The values of 4COL7s and HA were high in patients younger than one year of age, with no significant difference at older ages. Patients with high CVP also tended to have high 4COL7s values. Patients with high HA levels tended to have high CVP and low CI.

**Figure 3:**
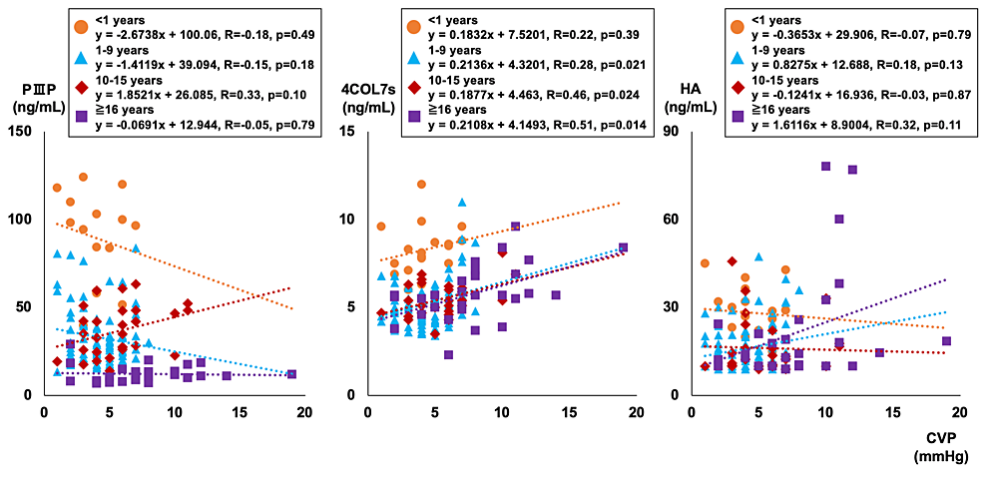
Correlations between CVP and liver fibrosis markers Orange circles are less than one year old, blue triangles are one to nine years old, red rhombuses are 10-15 years old, and purple squares are 16 years old or older. Three patients with abnormally high PIIIP (199, 208, 269ng/mL) were excluded from the graph. CVP: central venous pressure, PIIIP: procollagen type III peptide, 4COL7s: type IV collagen 7s, HA: hyaluronic acid

**Figure 4:**
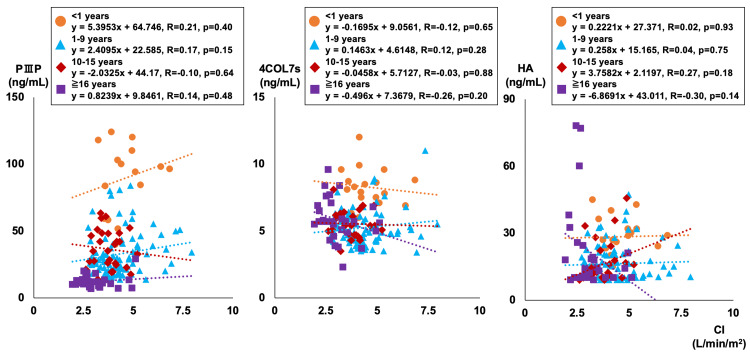
Correlations between CI and liver fibrosis markers Orange circles are less than one year old, blue triangles are one to nine years old, red rhombuses are 10-15 years old, and purple squares are 16 years old or older. Three patients with abnormally high PIIIP (199, 208, 269ng/mL) were excluded from the graph. CI: cardiac index, PIIIP: procollagen type III peptide, 4COL7s: type IV collagen 7s, HA: hyaluronic acid

 Figure 3 and Figure 4 show the correlation between CVP, CI, and liver fibrosis markers for each age group.

PⅢP and HA showed no significant correlation with CVP in any age group while 4COL7s showed a correlation with CVP in the age groups of one to nine years, 10-15 years, and 16 years and older (r = 0.28, r = 0.46, r = 0.51, respectively). No significant correlation was found with CI for any of the liver fibrosis markers.

## Discussion

In the present study, we found that in patients with CHD, all liver fibrosis markers were elevated in patients younger than one year of age, and PⅢP levels remained above the standard for adults in all age groups. 4COL7s showed a significant correlation with CVP in patients older than one year.

Adult patients with CHD are prone to hepatic congestion due to venous stasis and hepatic ischemia due to low cardiac output. Even in patients with Fontan circulation, chronic hepatic congestion and venous stasis associated with increased CVP are considered to be the main pathogenesis of Fontan-associated liver disease [11]. The present results suggest that elevated 4COL7s is more likely to be associated with elevated CVP, which may be due to hepatic congestion rather than hepatic ischemia. The relationship between 4COL7s and CVP has been previously reported, as 4COL7s is thought to reflect hepatic congestion at an early stage [4,12]. Although another report showed the benefit of measuring HA in adult patients after tetralogy of Fallot surgery, HA did not correlate well with CVP in our results [5].

It has been reported that PⅢP and HA are elevated in children under one year of age and in adolescents because of the growth-related development of connective tissues such as tendons, ligaments, and skin [13]. Therefore, elevated liver fibrosis markers in this age group do not directly correlate with hemodynamic abnormalities related to hepatic injury such as increased CVP. However, in recent years, the incidence of nonalcoholic fatty liver disease (NAFLD) in children has been increasing, and it is important to understand the fibrotic status of the liver in these patients [14]. In adolescent patients, some reports have found a cutoff PⅢP value of around 10 to be useful in diagnosing NAFLD [15]. However, CHD patients are prone to concomitant hepatic congestion and ischemia. In addition to cases of hepatic congestion, liver fibrosis markers have been reported to be elevated in cases of increased systemic-to-pulmonary blood flow ratios or myocardial fibrosis [16,17]. Although it is very important to monitor the fibrotic status of the liver in patients with CHD, it is difficult to set a predictive cutoff value for liver fibrosis markers.

However, based on our age-specific data, it may be appropriate to consider further testing for potential liver fibrosis when liver fibrosis markers deviate from the normal-for-age values. The three liver fibrosis markers examined in this study are early markers of disease and thus may detect subtle hepatic dysfunction. Testing for liver fibrosis markers may avoid unnecessary tests and may be cost-effective.

One of the limitations of this study was that liver function was assessed only by blood tests. A liver biopsy is necessary to accurately identify hepatic congestion and hepatic ischemia, but it cannot be performed in all patients because of its invasive nature. In the future, we plan to consider additional diagnostic modalities, such as magnetic resonance imaging and ultrasound with Doppler, to evaluate liver function in patients with high CVP.

## Conclusions

In this study, we found that elevated liver fibrosis markers, particularly 4COL7s, correlated with CVP in CHD patients older than one year. Routine measurement of liver fibrosis markers may allow early detection of changes in CVP and liver function in adult patients with CHD. Although it was difficult to correlate hemodynamic changes with liver fibrosis markers in patients aged under one year, the results of the present study allow us to create a similar standard for liver fibrosis markers in pediatric CHD patients, which will help in the treatment of liver pathology in children with CHD.

## References

[1] Møller S, Bernardi M (2013). Interactions of the heart and the liver. Eur Heart J.

[2] Wells ML, Fenstad ER, Poterucha JT (2016). Imaging findings of congestive hepatopathy. Radiographics.

[3] Yoshihisa A, Kimishima Y, Kiko T (2018). Liver fibrosis marker, 7S domain of collagen type IV, in patients with pre-capillary pulmonary hypertension. Int J Cardiol.

[4] Oka H, Nakau K, Imanishi R, Kajihama A, Azuma H (2021). Type IV collagen 7s reflects central venous pressure and right ventricular end-diastolic pressure in patients with congenital heart disease after biventricular repair. Pediatr Cardiol.

[5] Yamamura K, Sakamoto I, Morihana E (2019). Elevated non-invasive liver fibrosis markers and risk of liver carcinoma in adult patients after repair of tetralogy of Fallot. Int J Cardiol.

[6] Nakano H, Nakabayashi H, Okamoto Y, Kawasaki T, Seko S, Fukuda Y (1985). Serum levels of aminoterminal type III procollagen peptide in normal subjects and hepatic fibrosis. Gastroenterol Jpn.

[7] Erotokritou-Mulligan I, Bassett EE, Cowan DA, Bartlett C, McHugh C, Sönksen PH, Holt RI (2009). Influence of ethnicity on IGF-I and procollagen III peptide (P-III-P) in elite athletes and its effect on the ability to detect GH abuse. Clin Endocrinol (Oxf).

[8] Guha N, Erotokritou-Mulligan I, Burford C (2010). Serum insulin-like growth factor-I and pro-collagen type III N-terminal peptide in adolescent elite athletes: implications for the detection of growth hormone abuse in sport. J Clin Endocrinol Metab.

[9] Trivedi P, Cheeseman P, Mowat AP (1993). Serum hyaluronic acid in healthy infants and children and its value as a marker of progressive hepatobiliary disease starting in infancy. Clin Chim Acta.

[10] Nakazawa M, Marks RA, Isabel-Jones J, Jarmakani JM (1976). Right and left ventricular volume characteristics in children with pulmonary stenosis and intact ventricular septum. Circulation.

[11] Simonetto DA, Yang HY, Yin M (2015). Chronic passive venous congestion drives hepatic fibrogenesis via sinusoidal thrombosis and mechanical forces. Hepatology.

[12] Mizuno M, Shima T, Oya H (2017). Classification of patients with non-alcoholic fatty liver disease using rapid immunoassay of serum type IV collagen compared with liver histology and other fibrosis markers. Hepatol Res.

[13] Crofton PM, Wade JC, Taylor MR, Holland CV (1997). Serum concentrations of carboxyl-terminal propeptide of type I procollagen, amino-terminal propeptide of type III procollagen, cross-linked carboxyl-terminal telopeptide of type I collagen, and their interrelationships in schoolchildren. Clin Chem.

[14] Castillo-Leon E, Cioffi CE, Vos MB (2020). Perspectives on youth-onset nonalcoholic fatty liver disease. Endocrinol Diabetes Metab.

[15] Mosca A, Comparcola D, Romito I (2019). Plasma N-terminal propeptide of type III procollagen accurately predicts liver fibrosis severity in children with non-alcoholic fatty liver disease. Liver Int.

[16] Sugimoto M, Saiki H, Tamai A, Seki M, Inuzuka R, Masutani S, Senzaki H (2016). Ventricular fibrogenesis activity assessed by serum levels of procollagen type III N-terminal amino peptide during the staged Fontan procedure. J Thorac Cardiovasc Surg.

[17] Yamazawa H, Murakami T, Takeda A, Takei K, Furukawa T, Nakajima H (2015). Serum concentration of procollagen type III amino-terminal peptide is increased in patients with successfully repaired coarctation of the aorta with left ventricular hypertrophy. Pediatr Cardiol.

